# An etiological assessment of a deep vein thrombosis led to the discovery of a renal tumor collision: Case report

**DOI:** 10.1016/j.ijscr.2023.108922

**Published:** 2023-10-05

**Authors:** Hammou El Farhaoui, Anouar Elmoudane, Ahmed Jdaini, Abdessamad Moutaouakil, Ali Barki

**Affiliations:** Faculté de médicine et de pharmacie, Université Mohammed premier OUJDA, Morocco; Department of Urology, Centre Hospitalier Universitaire Mohammed VI - Oujda, Morocco

**Keywords:** Kidney tumor, Collision, Thrombosis, Case report

## Abstract

**Introduction and importance:**

The thromboembolic complication of kidney's tumor is rare, and they can be the reason for the discovery of those tumor. Also the collision kidney tumor, such as a simultaneous occurrence of different histological types of adjacent neoplasms in the same organ is rare.

**Case presentation:**

We report a patient diagnosed with a kidney tumor discovered in the context of an etiological assessment of thrombosis, presenting with pulmonary embolism and deep vein thrombosis of the lower limb. This tumor treated by a cytoreductive nephrectomy. The histologic diagnosis of PRCC (Papillary Renal Cell Carcinoma) associated with a chromophobe cell carcinoma and sarcomatoid component was rendered.

**Clinical discussion:**

The development of the tumor process and its progression to the metastatic stage is largely favored by the hypercoagulable state, and the cancer itself promotes the appearance of thrombo-enmbolic phenomena due to this phenomenon.

Two major studies recommend that immediate cytoreductive nephrectomy should be offered to metastatic patients with a good general condition.

**Conclusion:**

A renal tumor collision is rare, whereas the risk factors for a renal tumor collision are the same as a renal tumor without collision, just as the management of a metastatic renal tumor is the same. Understanding the thromboembolic physiopathology in the case of kidney cancer has made it possible to optimize management.

## Highlights

• the collision tumour is a simultaneous occurrence of different histological types of adjacent neoplasms in the same organ.
• kidney tumor collision is rare, it is reported in the literature 2 main types of collision either papillary carcinoma with chromophobe or onycocytoma
• thromboembolic complications are frequent for kidney tumors, but rarely as a reason for finding the kidney tumor

## 1 Introduction

A tumor collision in the kidney can present with the association of a papilary renal cell carcinoma with a chromophobe cell carcinoma [1] or the association of the papilary renal cell carcinoma with an oncocytoma [2]. A kidney tumor discovered by different symptoms more rarely by thrombosis, indeed the signs of deep vein thrombosis (DVT) or pulmonary embolism (PE) can be the mode of revelation of cancer [3] whose diagnosis is often radiological by scanner or MRI. Our rare case presents a renal tumor collision which must be mentioned in the literature given the rarity of the pathology as well as the discovered mode by thromboembolic symptom.

This case report has been reported in line with the SCARE Criteria [6].

## 2 Observation

### 2.1 Patient information

A 59-years-old patient, without no medical or surgical or toxic history, nor a family or personal history of tumors. He consults for a large right leg that has been evolving for 6 months without pain in the lumbar fossae or urinary signs like haematuria.

This symptom accompanied by dyspnea without cough or expectoration, which motivated the patient to consult, where he benefited from an ultrasound of the lower limb objectified a deep venous thrombosis.

Then he was transferred to the hospital for etiological assessment of this thrombosis.

### 2.2 Clinical findings

The examination revealed a fever of 40° with a dyspnea. The oxygen saturation in ambient air was normal, with a big right leg associated to a slight left lumbar sensitivity.

## 3 Diagnostic assessment

The biological assessment revealed a high level of inflammatory marks with high values of LDH and serum calcium with profound anemia. For this reason the patient is being put on a broad-spectrum antibiotic.

In front of this case of venous thrombosis associated with a fever, we evoked an embolism of infectious origin. The reason for which the patient benefited from a chest scan that found a pulmonary embolism and fortuitously a right renal mass associated with multiple lung lesions.

**Fig. 1:**
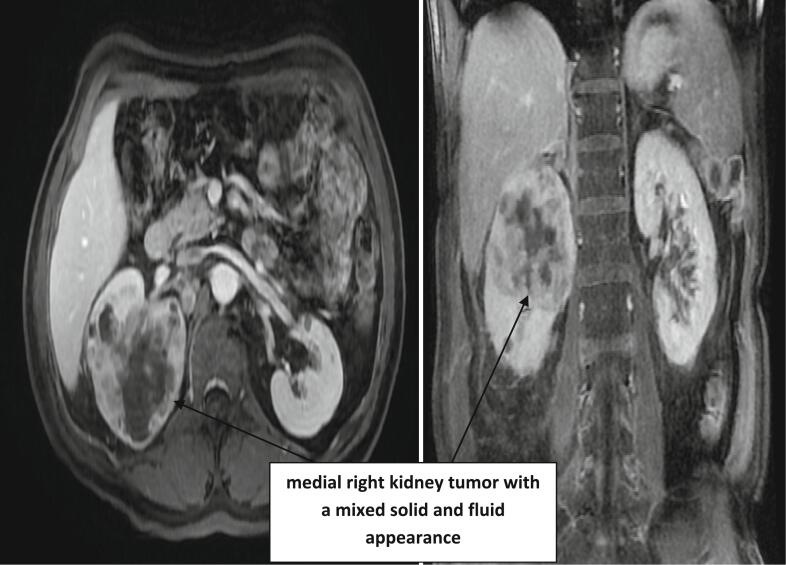
CT image of a right kidney tumor.

The scanner could not decide whether it is a renal tumor with pulmonary metastasis or pyelonephritis with pulmonary septic emboli. Hence the indication of the realization an MRI showing a heterogeneous upper pole right kidney mass measuring 81 cm with pulmonary metastasis (Fig. 1).

### 3.1 Therapeutic intervention

A total cytoreductive nephrectomy was performed for the patients by a surgeon and an operating assistant within 2 h without significant signs during the procedure. The surgical approach was a right subcostal laparotomy, the patient in dorsal decubitus, under general anesthesia with antibiotic prophylaxis based on second generation cephalosporin.

## 4 Follow-up and outcomes

The immediate postoperative course was favorable with no complications. The patient is put on curative anticoagulation and an analgesic.

**Fig. 2:**
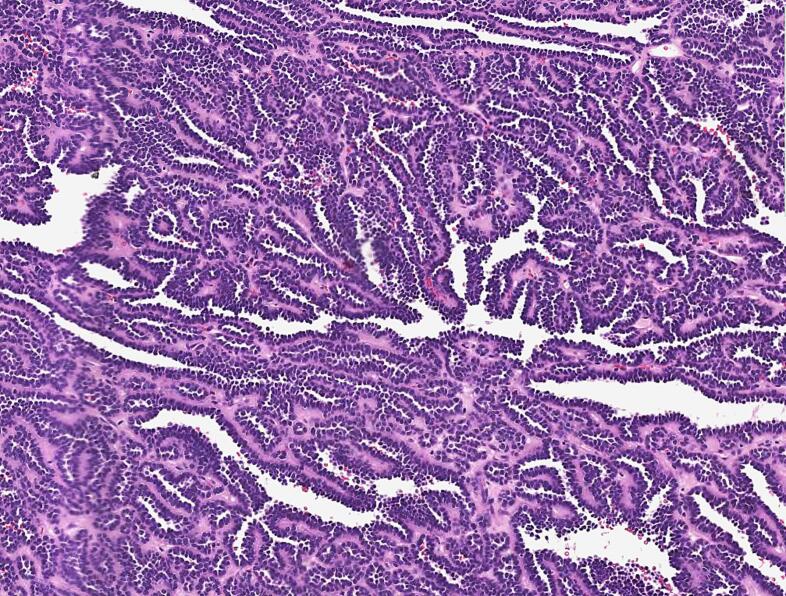
Microphotography of a first component (papillary renal cell carcinoma), made of papillary structures containing fibrovascular cores.

**Fig. 3:**
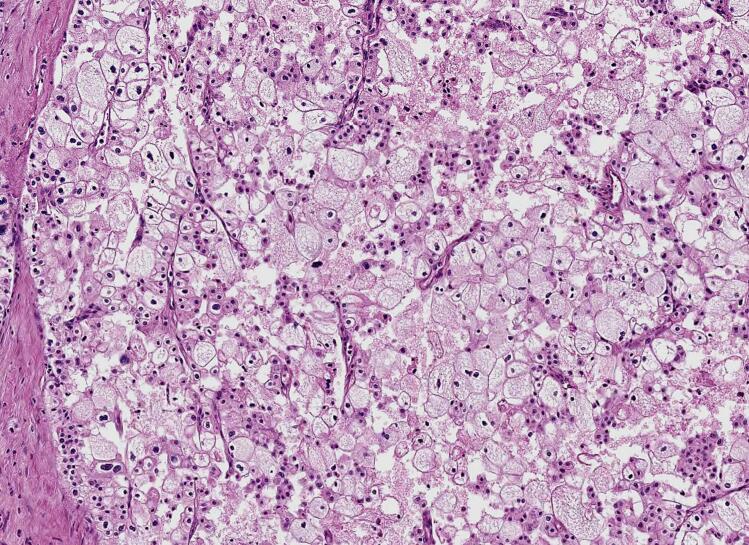
Microphotography of the second component (chromophobe renal cell carcinoma), showing a solid and nested growth. The cell have the characteristic clear perinuclear halo. (HE; 200×).

The histology diagnosis of papilary renal cell carcinoma associated with a chromophobe cell carcinoma was rendered (Fig. 2, Fig. 3).

Then the patient was sent to the oncology department for a possible immunotherapy project with a control by imaging in 3 months. The evolution of which was favorable by stability of the pulmonary metastases without the appearance of new lesions.

## 5 Discussion

Renal Cell Carcinoma is a heterogeneous tumor that includes different histology subtypes (based on morphology), including clear cell renal cell carcinoma, papilary renal cell carcinoma, Chromophobe Renal Cell Carcinoma and collection ductal carcinomas [1].

The presence of concurrent renal cell carcinoma in the same kidney as an oncocytoma has been described and most commonly involves clear cell renal cell carcinoma and chromophobe renal cell carcinoma. However, the occurrence of a papilary renal cell carcinoma and an oncocytoma within the same tumor mass is extremely rare and poorly known [2].

There are some cases reported in the literature such Zhang et al. in 2014 report a case of a 63-years-old woman wish a CT scans of the abdomen revealed two heterogeneous incidental right renal masses; Histopathological examination showed a collision tumor of the papillary renal cell carcinoma with chromophobe renal cell carcinoma [1]. Valderrama et al. report at first time in 1987 a case of a renal collision tumor comprising renal cell carcinoma (RCC) with squamous cell carcinoma (SCC) [4].

The association of venous thromboembolic disease and cancer is frequent. Also the thromboembolic risk is an important cause of morbidity and mortality in patients with cancer, the annual incidence is 4 to 7 times higher than that of the general population [3].

The development of the tumor process and in particular the metastatic switch is widely favored by the state of hypercoagulability [3]. The current recommendations all converge to say that long-term curative treatment of VTE can significantly reduce the risk of death and recurrence [3].for a good orientation of the management of metastatic kidney cancer the classification of the International Metastatic RCC Database Consortium (IMDC) is now the most used in clinical practice [5].

For the processing part, two studies recommended that an immediate cytoreductive nephrectomy can be offered to a metastatic patient in good general condition which does not require systemic treatment [5].

For localized tumors complete surgical excision may be the only curative treatment, and sorafenib is a better choice for adjuvant therapy. The few existing published case reports may not be sufficient to allow clinical outcome however, a longer follow-up period may be necessary to definitively assess clinical outcome renal collision [1].

## 6 Conclusion

A collision of renal tumors is rare, and rarely a collision of a papillary chromophobe carcinoma with an onycocytoma. Nephrectomy combined with long-term anticoagulant therapy will stabilize the tumor pathology. as well as the immunohistochemical characteristics of each subtype determine management and prognosis, hence the interest in immunotherapy.

## Consent

Written informed consent was obtained from the patients for publication and any accompanying images. A copy of the written consent is available for review by the Editor-in-Chief of this journal on request.

## Ethical approval

The ethical approval has been exempted for this type of article (case report) since it is not a human clinical trial, but just a presentation of a rare case as an added value to the literature, by our institution sources of funding: hospital management (Mohammed VI University Hospital, Oujda, Morocco).

## Sources of funding

There is no source of funding or sponsor for this article.

## Author contribution

The corresponding author **Hammou El Farhaoui HF** to participate in all stages of the research, **Anouar El Moudane AM** and **Ahmed Jdaini AJ** participated by research in the literature, **Abdessamad Moutaouakil AM** participated by the correction and formatting, this traiaville is supervised by **Ali Barki AB.**

## Guarantor

Hammou El Farhaoui

## Conflict of interest statement

All the authors hereby declare that no conflict of interest exists and there are no financial or personal relationships or affiliation that could influence (or bias) the author's decisions, work or manuscript.
